# Hydrothermal alteration of kimberlite by convective flows of external water

**DOI:** 10.1007/s00410-014-1038-y

**Published:** 2014-07-10

**Authors:** A. A. Afanasyev, O. Melnik, L. Porritt, J. C. Schumacher, R. S. J. Sparks

**Affiliations:** 1 Institute of Mechanics, Moscow State University, Moscow, Russia; 2 School of Earth Sciences, University of Bristol, Bristol, BS8 1RJ UK

**Keywords:** Kimberlites, Hydrothermal alteration, Serpentine

## Abstract

Kimberlite volcanism involves the emplacement of olivine-rich volcaniclastic deposits into volcanic vents or pipes. Kimberlite deposits are typically pervasively serpentinised as a result of the reaction of olivine and water within a temperature range of 130–400 °C or less. We present a model for the influx of ground water into hot kimberlite deposits coupled with progressive cooling and serpentisation. Large-pressure gradients cause influx and heating of water within the pipe with horizontal convergent flow in the host rock and along pipe margins, and upward flow within the pipe centre. Complete serpentisation is predicted for wide ranges of permeability of the host rocks and kimberlite deposits. For typical pipe dimensions, cooling times are centuries to a few millennia. Excess volume of serpentine results in filling of pore spaces, eventually inhibiting fluid flow. Fresh olivine is preserved in lithofacies with initial low porosity, and at the base of the pipe where deeper-level host rocks have low permeability, and the pipe is narrower leading to faster cooling. These predictions are consistent with fresh olivine and serpentine distribution in the Diavik A418 kimberlite pipe, (NWT, Canada) and with features of kimberlites of the Yakutian province in Russia affected by influx of ground water brines. Fast reactions and increases in the volume of solid products compared to the reactants result in self-sealing and low water–rock ratios (estimated at <0.2). Such low water–rock ratios result in only small changes in stable isotope compositions; for example, δO^18^ is predicted only to change slightly from mantle values. The model supports alteration of kimberlites predominantly by interactions with external non-magmatic fluids.

## Introduction

Many volcaniclastic kimberlite deposits are strongly altered with the original igneous minerals typically altered to hydrous minerals and carbonate. Serpentine is ubiquitously a major component of the alteration assemblage as a consequence of the high abundance of olivine in kimberlite. Fresh olivine is commonly not preserved (Mitchell 1986, 2013; Sparks 2013), and features such as serpentine pseudomorphing olivine, and serpentine replacing groundmass phases and precipitating in pore space, are observed. The origin of the water is controversial. Mitchell et al. (2009, 2013) have attributed the serpentine-dominated hydrous mineral assemblages to alteration related to deuteric (magmatic) fluids and regarded serpentine as a primary mineral. In contrast, others (Stripp et al. 2006; Hayman et al. 2009; Buse et al. 2011; Porritt et al. 2012; Giuliani et al. 2014) have argued that alteration is the result of circulation by external water through hot olivine-rich pyroclastic deposits. This controversy is addressed here through numerical modelling of post-emplacement external water circulation through kimberlite pipes. The focus here is on serpentinisation as the dominant reaction. The model couples fluid flow, heat transfer, and serpentinisation reactions with the evolution of porosity and permeability in the pipe’s interior. The model shows that strong influx of external water accompanied by hydrothermal metamorphism, represented in the model by serpentine formation, is inevitable. The distribution of kimberlite rocks that preserve fresh olivine is predicted and compared with observations from Diavik A418 kimberlite pipe (NWT, Canada) and also with features of kimberlites of the Yakutian province in Russia. External water is identified as the main cause of alteration on the basis of the models and other evidence. This study concerns volcaniclastic kimberlite deposits in diatremes and not hypabyssal kimberlites.

## Geological constraints and context

Kimberlite pipes are the conduits of monogenetic diatreme volcanoes (Lorenz 1975; Sparks et al. 2006; Scott Smith 2008). They are typically 1–3 km deep, steep-sided (~80^o^) and up to a few 100 m across. Idealised geological models developed in southern Africa (e.g. Hawthrone 1975) recognise a shallow crater zone, a downward tapering pipe, and a root zone, which can be wider than the overlying pipe. Subsequent studies have recognised a wider variety of kimberlite pipes (Field and Scott Smith 1999; Scott Smith 2008), including those that have wide craters and lack diatreme and root zones, and those that are infilled with reworked volcaniclastic deposits.

The geology of kimberlite pipes, summarised by Sparks (2013), indicates that the dominant lithologies infilling kimberlite pipes are volcaniclastic deposits. They typically display a wide diversity of different geological units and lithofacies, reflecting complex multiphase eruptions and a diversity of primary volcanic and secondary reworking processes. Layered and massive volcaniclastic rocks vary widely in grain size, sorting, and componentry; olivine (typically altered to serpentine) is a major constituent of most kimberlites. Contacts between different geological units range from steep near vertical and cross-cutting, to layer cake style. Layering is commonly steep with dips at or well above the angle of repose of loose volcaniclastic deposits, typically oriented towards the pipe centre. Important varieties of kimberlite pipe infill are dark coherent rocks, which have been described as hypabyssal, but more recently have been interpreted as high-temperature welded clastogenic rocks (Brown et al. 2008a). Kimberlite dykes and sills, which are volumetrically insignificant in pipes, are coherent rocks sensu stricto and more commonly preserve fresh olivine. Lithofacies analysis indicates some kimberlite eruptions occurred in a shallow submarine environment with extensive reworking (Pittari et al. 2008), whereas others occurred in sub-aerial settings. We discuss below the role of emplacement temperature in explaining the preservation of fresh olivine.

Recent investigations have highlighted that the emplacement temperatures of volcaniclastic kimberlites vary greatly and include both low-temperature and high-temperature varieties. Evidence for temperatures of up to several 100 °C includes the following: estimates from the thermal maturity of organic materials (Stasiuk et al. 1999); estimates from typical hydrothermal metamorphic mineral assemblages (Stripp et al. 2006; Buse et al. 2011); estimates of lithic clast temperatures from TRM studies (Fontana et al. 2011); and textural evidence of welding and agglutination of primary magma clasts (Brown et al. 2008a, 2009; van Straaten et al. 2011; Gernon et al. 2012). Low-temperature volcaniclastic deposits, likely related to quenching during phreatomagmatic styles of eruption, have also been recognised (Brown et al. 2008b; Kurszlaukis and Lorenz 2008; Pittari et al. 2008; Porritt et al. 2012; van Straaten and Kopylova 2013).

Host rocks to kimberlite vary greatly, but a fairly common setting is an Archaean basement overlain by younger sedimentary and volcanic strata; this arrangement is typical of many southern African and Canadian kimberlites.

Serpentine in kimberlites includes replacement of primary igneous minerals, principally olivine and components of the igneous groundmass, and precipitation of serpentine in pore space. Textural relationships show that there can be a multiple generations of serpentine (Mitchell 2013), and serpentine can be co-precipitated with other minerals such as diopside and chlorite (Stripp et al. 2006; Mitchell et al. 2009; Buse et al. 2011). Pervasive fracture networks, as commonly observed in serpentinised peridotite (Jamtveit and Austrheim 2010), are not observed in kimberlites. The large volumes changes of the serpentisation reaction are accommodated by filling of pore space, replacement of earlier minerals, and possibly open system loss of components rather than deformation.

## Conceptual model

**Fig. 1:**
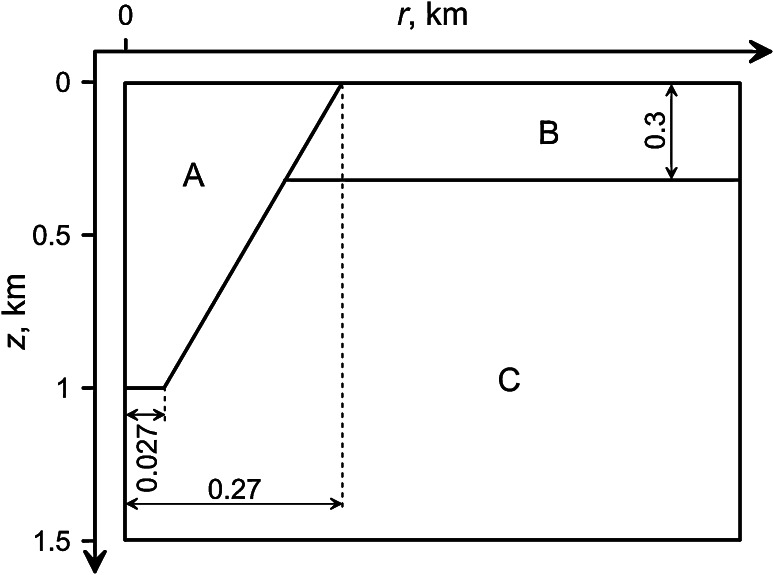
Schematic view of the system. Kimberlitic body (*A*) is a highly permeable porous conical pipe. Layers of the host rocks (*B*) and (*C*) can have different permeabilities and porosities. All dimensions are in km. Total horizontal extent of the domain is 2 km

The geological characteristics described above enable us to propose a conceptual and simplified hydrogeological model for investigation (Fig. 1). A kimberlite body is depicted as a downward tapering cone with depth 1 km and near surface width of 540 m (region A). In the simpler models, we assign a single set of properties to the host rocks. In more elaborate models, the surrounding strata embodies higher porosity and higher permeability rocks (region B) overlying low porosity and low permeability basement (region C). The kimberlite pipe is infilled with volcaniclastic deposits with an initial porosity and permeability greater than that of the host rocks. Here, we investigate the case where the pipe is infilled with hot volcaniclastic rocks at temperatures of several 100 °C. We examine cases where the initial porosity and permeability of the kimberlite are homogeneous and discuss the effects of geological complexities.

The model assumes that the host rocks are saturated with water. The emplacement of the kimberlite is assumed to be very short compared to typical cooling times of the kimberlite pipe and so is simplified to being instantaneous. This assumption will be justified a posteriori. The temperature is high with pores occupied by low-density volcanic volatiles. The initial pore pressure inside the kimberlite is assumed to be very low and approximately atmospheric. Another way of describing the last assumption is that the kimberlite has sufficiently high porosity and high permeability that it is well connected to the atmosphere above. The volcaniclastic deposits that infill kimberlite pipes are inferred to have similar high permeabilities to other kinds of moderately to poorly sorted pyroclastic deposits (Wilson 1984; Miller 1990). If accumulation rates are high, then the deposits might develop transient pore pressures approaching lithostatic values. These pore pressures, however, dissipate rapidly. Scaling analysis (Sparks et al. 1999) allows calculation of an approximate time scale *τ* ≈ *H*/(*Kp*_*z*_/*μ*_g_), where *K* is permeability, *p*_*z*_ is the pressure gradient across the layer, and *μ*_g_ is the gas viscosity. For *H* = 1 km (a typical pipe depth), *μ*_g_ = 10^−5^ Pa s, *K* = 10^−12^ m^−2^ (a representative permeability), and *p*_*z*_ = 2 × 10^4^ Pa m^−1^ (a representative lithostatic gradient), and the time scale *τ* is approximately 1 month to be compared with a typical cooling time in the order of 1,000 years.

With these assumptions, a large horizontal pressure gradient is initially present between the hydrostatic pressure in the host rock and the low pressure in the kimberlite, resulting in water being drawn into the pipe interior along horizontal flow paths. Here, the water is heated and mostly flows upwards in the pipe interior driven by large vertical pressure gradients. The water reacts with the olivine to form serpentine. Besides olivine, other primary igneous minerals, possible glass, and incorporated accidental lithic clasts are reactive and other alteration minerals may form (e.g. talc and chlorite). However, the serpentisation reaction is quite well understood (see below), and olivine is a major constituent, so first-order features of the alteration process can be captured by a model in which only the serpentisation is considered.

Serpentinisation involves a large volume increase, and here, it is assumed that this volume is locally divided between replacing olivine and infilling pore space. With the exception of the addition of water, we model the process as chemically closed with no constituents derived from the original unaltered kimberlite being transported out of the system. Mg and Si are regarded as immobile on a large scale, but locally can be mobile on the scale of pores to facilitate serpentisation reactions. As water flows through and reacts with the kimberlite, heat is generated due to the exothermic character of the reaction. The porosity and permeability of the kimberlite decrease with time as pore space are filled with serpentine.

The process of serpentinisation of ultramafic rocks has mostly been studied in peridotite bodies. The serpentinisation reactions and the conditions in kimberlites share some attributes with those of serpentinisation of other ultramafic bodies, but key differences exist. Kimberlites are rich in olivine and other low-silica primary minerals, and possible glass or melt may be present at emplacement when temperatures are high. The dynamics of emplacement results in the admixture of the primary kimberlitic constituents with xenoliths of other crustal rock types, which are richer in silica. The volcaniclastic deposits of the kimberlite pipes can have an initial porosity of 20–30 % (e.g. Stripp et al. 2006). Fracturing of the country rock that accompanies the emplacement of the cross-cutting kimberlite pipe will likely intersect aquifers, which can deliver large amounts of groundwater to the hot kimberlite pipe and initiate serpentine formation from olivine in addition to phreatic activity.

The mechanism of olivine hydration in kimberlites and other ultramafic bodies is commonly given by the end-member reaction:

1 $$\mathop{{2{\rm{ M}}{{\rm{g}}_2}{\rm{Si}}{{\rm{O}}_4}}}\limits_{{\rm{olivine}}} + \mathop{{3{\rm{ }}{{\rm{H}}_2}{\rm{O}}}}\limits_{{\rm{fluid}}} \Rightarrow \mathop{ {{\rm{M}}{{\rm{g}}_3}{\rm{S}}{{\rm{i}}_2}{{\rm{O}}_5}{{\left( {{\rm{OH}}} \right)}_4}}}\limits_{{\rm{serpentine}}} + \mathop{{{\rm{Mg}}{{\left( {{\rm{OH}}} \right)}_2}.}}\limits_ {{\rm{brucite}}}$$

Access of water-rich fluid is a pre-condition for serpentine formation. Olivine will remain completely unaltered or partially to completely converted to hydrous phases by various reactions depending on the presence and access of water. For rocks that are not fully hydrated, i.e. olivine-bearing rocks, influx of water at temperatures below the equilibrium P–T conditions of the serpentine-forming reactions will still result in conversion of olivine by the stoichiometry of the appropriate reaction. As long as temperatures are high enough that the reactions are not inhibited by kinetics, these reactions can occur over a range of temperatures within the serpentine stability field. For large ultramafic bodies, Frost and Beard (2007) suggest serpentinisation will be controlled by fluid access, and when *P*_H2O_ < *P*_total_, serpentinisation could begin well below the temperature of reaction (1) via the following:

2 $$\mathop{3 {\text{ Mg}}_{2} {\text{SiO}}_{ 4}}\limits_{{\text{olivine}}}\,+\,1 {\text{ SiO}}_{ 2} \left( {\text{aq}} \right) \,+\, \mathop{4 {\text{ H}}_{2} {\text{O}}}\limits_{\text{fluid}} \Rightarrow \mathop{2 {\text{ Mg}}_{ 3} {\text{Si}}_{ 2} {\text{O}}_{ 5} \left( {\text{OH}} \right)_{ 4}}\limits_{{\text{ serpentine}}}.$$

In ultramafic bodies, reaction (2) would rapidly deplete the fluid in silica leading to the production of brucite (Frost and Beard 2007). In kimberlites, reaction mechanisms similar to reaction (2) are suggested by apparent isovolumetric replacement of olivine by serpentine (Stripp et al. 2006). These textures seem to require local mobility of MgO in addition to SiO_2_, and the redistribution of MgO is supported by texturally different serpentine precipitating in pore spaces near and around the locations of isovolumetric olivine replacement (Stripp et al. 2006). The fayalite component of olivine can provide a source of silica through the formation of magnetite, combining with excess Mg implied by reaction (1) and providing some of the silica implied in reaction (2). In kimberlites, the presence of admixed xenolithic country rock fragments also provides a silica rich source distributed within the volcaniclastic kimberlite (e.g. Caro et al. 2004; Buse et al. 2011) that can facilitate serpentinisation via reactions similar to reaction 2.

Serpentine-forming reactions are exothermic (i.e. heat of reaction, ∆*H* is given by the products minus the reactants is negative); however, the amount of heat produced is dependent on the reaction path and progress. Complete conversion of forsterite via reaction (1) has a ∆*H* = −352 kJ/kg, while for reaction (2), ∆*H* = −326 kJ/kg (e.g. values from Robie et al. 1979). Fyfe and Lonsdale (1981) estimated heat production of about 260 kJ/kg from the serpentinisation of ocean-floor ultramafic rocks. Their value is lower than values estimated from Mg end members (reactions 1 and 2 above). This discrepancy in heat production may in part be due to the fayalite component of the olivine. During serpentinisation, the fayalite component is commonly converted to magnetite + aqueous silica via the reaction:

3 $$\mathop{3 {\text{Fe}}_{ 2} {\text{SiO}}_{ 4}}\limits_{\text{fayalite}} + \mathop{2 {\text{ H}}_{ 2} {\text{O}}}\limits_{{\text{fluid}}} \Rightarrow \mathop{2 {\text{ FeFe}}_{ 2} {\text{O}}_{ 4}}\limits_{{\text{magnetite}}} +\mathop{ 3 {\text{ SiO}}_{ 2} \, \left( {\text{aq}} \right) \, + {\text{ H}}_{ 2}.}$$

This reaction (3) is endothermic reaction with ∆*H* = 299 kJ/kg. As a consequence, conversion of olivine with mantle composition (fo_92_fa_08_) to serpentine + magnetite would give ∆*H* = −300 kJ/kg for combined reactions 1 and 3 and ∆*H* = −276 kJ/kg for combined reactions 2 and 3, which is very close to estimated heat production of about 260 kJ/kg estimated for serpentinisation by Fyfe and Lonsdale (1981).

**Fig. 2:**
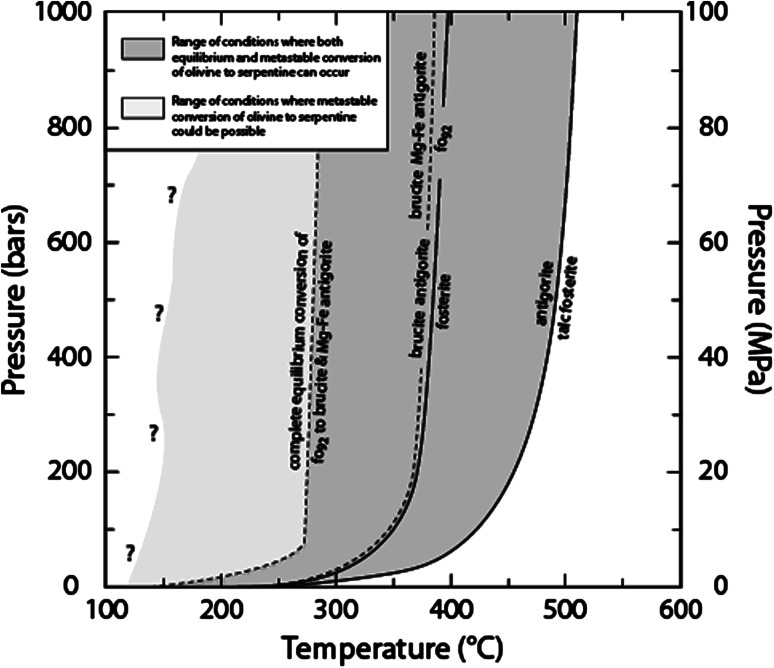
Pressure–temperature diagram showing the limits on stability of forsterite and antigorite in the MgO–SiO_2_–H_2_O system (*solid lines*) and part of an isochemical P–T section showing the equilibrium conversion of olivine (fo_92_) to Mg–Fe serpentine (*dashed lines*). The olivine (fo_92_) composition (CaO:0.02–FeO:8.11–MnO:0.12–MgO:50.37–SiO_2_:40.34) is the average of eight analyses of kimberlitic olivine grains (Table 1, Kamenetsky et al. 2004), and P–T range of equilibrium conversion to serpentine was calculated with Perple_X (Connolly 2005) assuming H_2_O saturation. (*Question mark*) indicates uncertainty of the lower thermal boundary of metastable conversion of olivine to serpentine

Reaction (1) occurs at approximately 400 °C, and the upper thermal stability of serpentine (antigorite) lies near 500 °C and is limited by the assemblage forsterite + talc at fluid saturated conditions (Fig. 2). These end-member stability fields suggest a range of about 400–500 °C antigorite + forsterite. Using a representative kimberlitic, olivine (fo_92_fa_08_) composition extends the range of P–T conditions for the equilibrium conversion of a kimberlitic olivine + water to Mg–Fe serpentine to about 275 °C. Because access of water to olivine is critical, serpentine-forming reactions (Fig. 2) may begin and progress at temperatures well below the stability field of olivine + water, if fluid ingress occurs at these lower temperatures. This metastable conversion of olivine to serpentine, e.g. via reactions 2 and 3, could occur at temperatures below 275 °C.

**Fig. 3:**
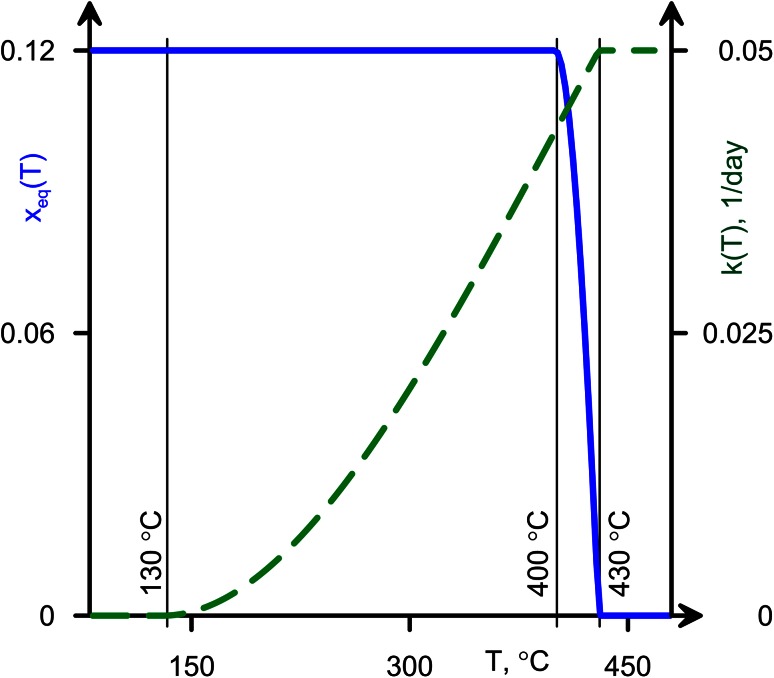
Parameters of the kinetic equation. Equilibrium mass fraction of bonded water (*blue curve*) and reversed reaction time (*dashed green*) is shown as a function of the temperature. We assume that serpentinization becomes insignificant for temperatures below 130 °C

The kinetics of serpentinisation is strongly temperature-dependent. Evans et al. (2013) indicate that the progress of serpentinisation reactions can be measured at the time scales of laboratory experiments, may continue at temperatures 50–60 °C below equilibrium conditions, and show sizeable volumetric changes over days or weeks. Along these lines, MacDonald and Fyfe (1985) suggested 1 km^3^ of peridotite could be serpentinised in 1,000 years at 300 °C, which suggests rapid reaction rates, and based on their experimental data and the literature. MacDonald and Fyfe (1985) suggest rates of serpentinisation should be rapid at temperatures above about 100 °C. Figure 3 shows a representative kinetic model for serpentinisation used in the calculations that will be discussed later.

## Mathematical model

In order to simulate cooling of a kimberlite body by external water influx, we have used a modified version of the filtration code MUFITS (Afanasyev 2013a, b). The code is developed for simulation of multiphase multicomponent flows in porous media in a wide range of pressures and temperatures, including sub-critical and supercritical conditions. It solves mass conservation laws for individual components (water and CO_2_) together with energy equation for the system as a whole including the solid rock matrix, and Darcy transport equations for different phases. The equation of state is specified in a table form of spline coefficients for the thermodynamic potential (entropy) that allows calculation of all properties of the mixture in terms of pressure, enthalpy, and total composition. Two modifications of the code were implemented: Serpentinisation of the olivine leads to a decrease in the density of the rock matrix and filling pore spaces resulting in significant decrease in porosity and permeability; latent heat of serpentinisation is accounted for in the energy equation.

### Kimberlite and host rock properties

Manning and Ingebritsen (1999) summarised estimates of the bulk permeability of continental crust. They show that in the uppermost 5 km of the crust, the permeability varies between 10^−12^ and 10^−16^ m^2^. We thus have chosen a range of host rock permeabilities between 10^−15^ and 10^−16^ m^2^. Volcaniclastic kimberlites have similar sorting and grain size to many pyroclastic deposits. They are expected to have similar permeabilites to unconsolidated to weakly indurated pyroclastic flow deposits, which have measured permeabilities in the range 3 × 10^−13^–3 × 10^−15^ m^2^ (Wilson 1984; Miller 1990; Sruoga et al. 2004). We use an illustrative ratio of initial kimberlite to host rock permeability of 300 with initial kimberlite permeability of 3 × 10^−14^ m^2^. Values for particular calculations are given in the figures displaying the results.

We assume that initially pipe-filling kimberlite deposit consists of two types of material: non-reactive (inert) fragments and olivine. The non-reactive material includes igneous minerals other than olivine and country rock xenoliths, which are treated as non-reactive in this simplified model, although we recognise that in nature, some of these materials are likely to also hydrate. Our goal here though is to develop an alteration model based on the conversion of olivine to serpentine that occurs in the presence of water. The amount of bound water in the rock matrix determines the degree of serpentinisation. While other reactive constituents could be modelled by similar algorithms, their inclusion is not expected to produce significantly different results because they also require similar amounts of water and involve comparable volume changes. Thus, at first order, they can be considered as being represented by the serpentinisation of olivine.

We consider the absolute densities, *ρ,* of the solid phases to be constant and the specific internal energies to be linear functions of the temperature:

4 $$\rho_{i} = {\text{const}},\quad e_{i} = \tilde{e}_{i} + C_{i} (T - T_{e} ),\quad i = {\text{in}},{\text{ol}},{^*}.$$

Here, index (in) refers to inert components, the index (ol) refers to the olivine, the index (*) refers to the bound water, *C*_*i*_ is the specific heat capacity, *T* is the temperature, and $\tilde{e}_{i}$, *T*_*e*_ are constants.

We introduce the following primary variables, which define the composition of the kimberlitic rock:

5 $$x = \frac{{M_{ * } }}{{M_{\text{ol}} + M_{ * } }};\quad y = \frac{{M_{\text{ol}} }}{{M_{\text{ol}} + M_{\text{in}} }} ,$$

where *M* is the constituent mass in an elementary volume, *x* is the mass fraction of the water bound within serpentine, and *y* is the initial mass fraction of olivine in the kimberlitic rock before the alteration.

Using the variables (5), we derive the relations for mass fraction of the three constituents:

6 $$z_{\text{in}} = \frac{(1 - x)(1 - y)}{1 - x + xy};\quad z_{\text{ol}} = \frac{(1 - x)y}{1 - x + xy};\quad z_{ * } = \frac{xy}{1 - x + xy},$$

where *z*_*i*_, *i* = in, ol,* are the phase mass fractions of inert component, olivine, and bound water. We do not introduce a separate serpentine fraction because it can be calculated directly from the mass balance using three other components. The mass fractions of all three phases sum to unity:

7 $$z_{\text{in}} + z_{\text{ol}} + z_{*} = 1,$$

The bulk density of the rock matrix of kimberlitic deposit *ρ*_*s*_ is given by the relation:

8 $$\rho_{s} = (1 - x + xy)\left( {\frac{(1 - x)(1 - y)}{{\rho_{\text{in}} }} + \frac{y(1 - x)}{{\rho_{\text{ol}} }} + \frac{xy}{{\rho_{*} }}} \right)^{ - 1}.$$

According to (8), the deposit density prior the alteration (*x* = 0) is equal to

9 $$\rho_{s0} = \left( {\frac{1 - y}{{\rho_{\text{in}} }} + \frac{y}{{\rho_{\text{ol}} }}} \right)^{ - 1},$$

where index 0 refers to the parameters prior alteration.

The specific energy of the kimberlite deposit is given by the equation:

10 $$e_{s} = z_{\text{in}} e_{\text{in}} + z_{\text{ol}} e_{\text{ol}} + z_{ * } e_{ * } .$$

The thermophysical parameters of inert components, olivine, and bound water are summarised in Table 1.

**Table 1:** Parameters of kimberlitic rock

Parameter	Value
Bounded water density ρ_*_	713.8 kg/m^3^
Bounded water heat capacity C_*_	3 kJ/(kg K)
Heat of reaction *q*_*e*_	260 kJ/kg
Maximal bonded water content *x*_*e*_	0.12
Olivine density *ρ*_ol_	3,300 kg/m^3^
Olivine heat capacity *C*_ol_	1 kJ/(kg K)
Inert component density *ρ*_in_	2,900 kg/m^3^
Inert component heat capacity *C*_in_	0.84 kJ/(kg K)
Minimal porosity *m*_min_	0.01
Pressure *P*_*e*_	10 MPa
Temperature *T*_*e*_	423.15 K

### Serpentinisation of olivine

Serpentinisation reactions result in the reconstructive hydration of olivine to form serpentine polymorphs. In order to account for changes in the density due to the structural bounding of water in serpentine, we use the definition of the density of a multiphase mixture through mass fraction of its components:

$$\rho_{\text{serp}} = \left( {\frac{1 - x}{{\rho_{\text{ol}} }} + \frac{x}{{\rho_{*} }}} \right)^{ - 1} .$$

Here, *ρ*_serp_ is the density of the serpentine. The stoichiometry of serpentine gives *x* = 0.12 (Eq. 5). Assuming *ρ*_serp_ = 2,300 kg/m^3^ and *ρ*_ol_ = 3,300 kg/m^3^, the effective density of the water bonded within serpentine is ρ_*_ = 713.8 kg/m^3^. Water bound within serpentine is treated as a separate phase in the model as this simplifies calculations of mass balance for water in a porous media. By analogous methods, bound water heat capacity C_*_ = 3 kJ/kg/K is determined.

The heat of serpentinisation is defined by the constant $\tilde{e}_{ * }$ in the Eq. (4). We need to specify this constant in accordance with the specific enthalpy of water in free form in order to have a correct value for the heat of serpentinisation. The specific enthalpy of the bounded water must be less than the specific enthalpy of water in free form. Thus, if water transits from free to bounded form, its enthalpy decreases. The released heat of serpentinisation is the difference of the enthalpies (Landau and Lifshitz 1951). The enthalpy *h* of each phase is defined as

$$h_{i} = e_{i} + P/\rho_{i} ,\quad i = {\text{in}}, {\text{ol}}, *.$$

Following Fyfe and Lonsdale (1981), we use the value for the heat of serpentinisation *q*_*e*_ = 260 kJ/kg. In terms of the model, we can write the following energy conservation equation:

11 $$M_{\text{ol}} h_{\text{ol}} (P_{e} ,T_{e} ) + M_{ * } h_{w} (P_{e} ,T_{e} ) = M_{\text{ol}} h_{\text{ol}} (P_{e} ,T_{e} ) + M_{ * } h_{ * } (P_{e} ,T_{e} ) + (M_{\text{ol}} + M_{*} )q_{e}.$$

Here, *h*_w_ is the enthalpy of pure water in free form and *P*_*e*_ is a constant. The left-hand side of the relation (11) is the enthalpy of the reactants before serpentinisation, whereas the right-hand side is the enthalpy after the serpentinisation. The last term on the right-hand side is the released heat of serpentinisation. In this equation, we assume that all water participates in the reaction. Thus, using (5) and (11), the enthalpy of bounded water can be determined as follows:

$$\tilde{e}_{ * } = h_{\text{w}} (P_{e} ,T_{e} ) - \frac{{P_{e} }}{{\rho_{ * } }} - \frac{{q_{e} }}{{x_{e} }}.$$

Here, *x*_*e*_ = 0.12 is the mass fraction of the bounded water after complete serpentinisation.

### Alteration of the petrophysical parameters

The serpentinisation process in porous kimberlite deposits results in the porosity and absolute permeability reduction because the specific volume of the serpentine is larger than that of the olivine. The mass conservation law for the olivine phase can be represented as follows:

12 $$(1 - m_{0} )\left. {\left( {\rho_{s} z_{\text{ol}} } \right)} \right|_{x = 0} = (1 - m)\rho_{s} z_{\text{ol}} .$$

Here, *m* is the porosity. The left-hand side of the Eq. (12) is the bulk olivine density before alteration, while the right-hand side is the bulk olivine density after alteration.

We suppose that the absolute densities of olivine and inert components are constant. From the Eqs. (3)–(8) and (12), we can derive the relation for the porosity depending on the primary variables *x* and *y*:

13 $$m = 1 - (1 - m_{0} )\left\{ {1 + \frac{x}{1 - x}\frac{{y\rho_{s0} }}{{\rho_{ * } }}} \right\} .$$

According to the Eq. (13), the porosity *m* decreases if the fraction of bounded water *x* increases. In general, the porosity can become negative if *x* is large enough. We introduce the minimal porosity *m*_min_ to eliminate this unphysical situation. We assume that the serpentinisation reaction stops if the porosity *m* reaches the minimal value *m*_min_. Using the Eq. (13), we can calculate the maximum fraction of the bounded water *x*_max_ for which the minimal porosity *m*_min_ is reached:

$$x_{\hbox{max} } = \frac{{m_{0} - m_{\hbox{min} } }}{{1 - m_{0} }}\left( {\frac{{y\rho_{s0} }}{{\rho_{ * } }} + \frac{{m_{0} - m_{\hbox{min} } }}{{1 - m_{0} }}} \right)^{ - 1} .$$

Therefore, the serpentinisation progresses only if *x* ≤ *x*_max_. If the fraction of bounded water *x* reaches the maximal value *x*_max_, the reaction stops.

The absolute permeability *K* is assumed to be the following function of the porosity:

14 $$\frac{K}{{K_{0} }} = \left( {\frac{m}{{m_{0} }}} \right)^{3} ,$$

where *K*_0_ is the permeability before alteration.

### Reaction equation

We define serpentinisation kinetics by the equation:

15 $$\frac{{{\text{d}}x}}{{{\text{d}}t}} = k(T)\frac{{\rho_{\text{w}} }}{{\rho_{\text{w}}^{0} }}\left( {\hbox{min} (x_{\text{eq}} (T),x_{\hbox{max} } ) - x} \right)$$

Here, *t* is the time, *k*(*T*) is the reaction rate, and *x*_eq_(*T*) is the bounded water fraction in thermodynamic equilibrium with serpentine, *ρ*_w_ is the effective water density in the mixture, and *ρ*_w_^0^ = 1,000 *kg*/*m* ^3^ is a constant.

At low water pressures, serpentinisation occurs over an intermediate range of temperatures *T* ∊ [*T*_*α*_, *T*_*β*_] (Fig. 3), where we assume *T*_*α*_ = 130 °C, *T*_*β*_ = 430 °C. If *T* > *T*_*β*_, then the equilibrium value *x*_eq_ is equal to zero leading to zero rate of the serpentinisation. This temperature range is arbitrary, but lies within the stability field of serpentine minerals. The *T*_*α*_ used here is slightly above 100 °C, which MacDonald and Fyfe (1985) give as the lower limit of fairly rapid serpentinisation. We assume that in a narrow range of temperatures, the equilibrium bonded water fraction *x*_eq_ increases up to its maximum value with decreasing temperature and stays constant at lower temperatures.

The characteristic time of reaction *k* ^−1^(*T*) at *T* = *T*_*β*_ is approximately 20 days (Evans et al. 2013). As the temperature decreases, the reaction time tends to infinity and the reaction rate tends to zero (Fig. 3). This kinetics is oversimplified in many senses. However, as the timescale of kimberlitic pipe cooling is much longer than the reaction timescale, the system will stay close to equilibrium until the temperature decreases significantly, leading to an increase in the kinetic timescale.

### Hydrodynamics

We use the model for binary mixture flows in porous media (Afanasyev 2013a) for modelling the hydrodynamic processes associated with meteoric water invasion. The pipe-filling kimberlite deposits can be saturated by a binary mixture of water and a passive fluid component which does not participate in serpentinisation (i.e. air). Because the model of Afanasyev (2013a) is developed for water–CO_2_ mixtures instead of air, we use CO_2_ as the passive fluid component. This assumption is consistent with the widespread view that CO_2_ is major component of the volatile component associated with kimberlite magma (reviewed in Sparks 2013).

We use two mass balance equations for passive fluid component (16), water (17) and energy balance (18), which account for both the convective heat transfer and heat conduction. We also use the multiphase Darcy law (19) with relative permeability curves *f*_*i*_ as given in Brooks and Corey (1964). The hydrodynamic equations used in the present study are described in detail in Afanasyev (2013a, b). The rock density and specific energy are calculated by the relations (8) and (10), and the density of the water is the sum of two terms representing free and bounded forms.

16 $$\frac{\partial }{\partial t}\left( {m\sum\limits_{i}^{{}} {\rho_{i} c_{i(1)} s_{i} } } \right) + \text{div} \left( {\sum\limits_{i}^{{}} {\rho_{i} c_{i(1)} {\mathbf{w}}_{i} } } \right) = 0 ,$$

17 $$\frac{\partial }{\partial t}\left( {m\sum\limits_{i}^{{}} {\rho_{i} c_{i(2)} s_{i} } + (1 - m)z_{ * } \rho_{s} } \right) + \text{div} \left( {\sum\limits_{i}^{{}} {\rho_{i} c_{i(2)} {\mathbf{w}}_{i} } } \right) = 0 ,$$

18 $$\frac{\partial }{\partial t}\left( {m\sum\limits_{i}^{{}} {\rho_{i} e_{i} s_{i} } + (1 - m)\rho_{s} e_{s} } \right) + \text{div} \left( {\sum\limits_{i}^{{}} {\rho_{i} h_{i} {\mathbf{w}}_{i} } - (1 - m)\lambda_{s} {\mathbf{grad}}\,T} \right) = 0 ,$$

19 $${\varvec{w}}_{i} = - K\frac{{f_{i} }}{{\mu_{i} }}\left( {{\mathbf{grad}}\,P - \rho_{i} {\mathbf{g}}} \right) .$$

Here, index *i* = 1, 2, 3 refers to a binary mixture phase, index *s* refers to the rock properties, *c*_*i*(*j*)_ is the mass fraction of the *j*th component in the *i*th phase (*j* = 1 for CO_2_, *j* = 2 for water), *s*_*i*_ is the saturation, ***w***_*i*_ is the Darcy velocity, *e*_*i*_ is the internal energy, *P* is the pressure, *K* is the absolute permeability, *μ*_*i*_ is the viscosity, *f*_*i*_ is the relative permeability, and *λ* is the heat conduction coefficient.

The effective water density *ρ*_w_ in the Eq. (15) is calculated as

$$\rho_{\text{w}} = \sum _{i} \rho_{i} c_{i(2)} s_{i} .$$

## Results

**Fig. 4:**
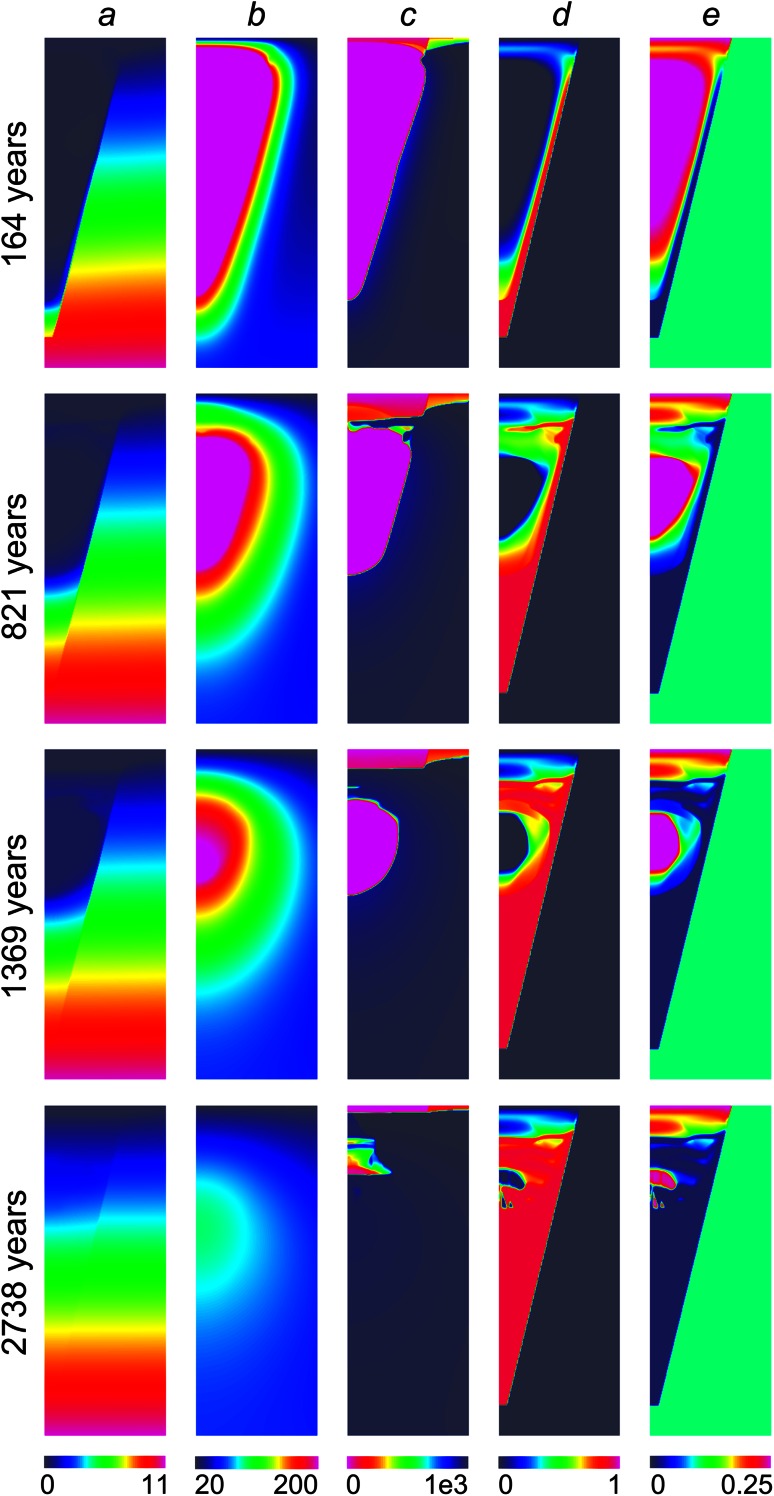
Snapshots of time evolution of the **a** pressure (MPa), **b** temperature (K), **c** bulk water density (kg/m^3^), **d** fraction of serpentinised olivine, and **e** porosity for pipe and host rock permeabilities of 3 × 10^−14^ m^2^ and 10^−16^ m^2^

Figure 4 shows the evolution of the temperature, pressure, water content, and the fraction of serpentinised olivine with time for the parameters of the system with properties summarised in Table 1, for the case when the permeabilities of regions B and C (*K*_*B*_ and *K*_*C*_) are equal to 10^−16^ m^2^. The pipe permeability *K*_*A*_ is taken to be 300 times higher than that of the host rock (3 × 10^−14^ m^2^). Initially, the pipe is filled with CO_2_, which is used as a proxy for air or residual volcanic gases, and has a temperature of 900 K. After a short period of time (~3 years), the initial hydrostatic pressure distribution evolves to the pressure distribution that is governed by water entrainment. On a time scale that is orders of magnitude shorter than the pipe cooling time, the influence of initial conditions on the solution vanishes and the pressure evolution is controlled by internal dynamics of the pipe cooling that is described by the model developed here. The pressure distributions inside the pipe and in the host rock are then close to hydrostatic, but, because the pipe is filled with hot gas connected to the atmosphere, pore pressure inside the pipe is initially close to atmospheric (Fig. 4a). The pressure in the rock has the value of 10 MPa outside the bottom of the pipe. The hydrostatic pressure in the groundwater increases with depth, creating a large horizontal pressure gradient, driving water into the pipe. Fluid moves into the pipe mostly from the bottom (see Fig. 4c). The temperature evolution (Fig. 4b) is controlled by convective cooling due to ingress of cold water and by conduction from the sides and the top of the pipe. Upon cooling, the upper central part of the pipe, furthest from the pipe margins, remains hottest longest. Results of the simulation show that conduction is the dominant heat transfer mechanism, because the water/rock ratio remains small during cooling meaning convective heat loss is low. Implication of low water/rock ratio on the oxygen isotope ration will be discussed later.

The uppermost part of the kimberlite cools down quickly due to heat loss to the atmosphere, and the temperature rapidly drops to below *T*_*α*_ = 130 °C suppressing serpentinisation. This leads to the formation of a high-permeability channel at the top of the pipe, enabling intense water influx in this region (see Fig. 4c). The water front in the deep part of the kimberlite is sharp because the temperatures are high enough that most of the water immediately reacts to form serpentine. A region filled with CO_2_ is isolated in the centre of the pipe suppressing serpentinisation (e.g. Fig. 4c, 1,370 years). Competition of conductive cooling and water inflow into the pipe leads to a complicated pattern of serpentine distribution because the reaction occurs over a relatively narrow range of temperatures in water-saturated conditions. Serpentinisation ceases after 1,860 years when the temperature within the entire pipe drops below *T*_*α*_ = 130 °C as shown in Fig. 3.

The process of serpentinisation is strongly controlled by the evolution of the porosity and permeability (Eq. 12). Serpentinisation causes a large increase in the volume of the matrix of the kimberlite, reducing water flux through the serpentinised part of the pipe. Reduction of permeability along the pipe margins in the early stages of the cooling results in decrease in water flux into the system and leads to predominantly conductive cooling of the pipe. Only the upper part of the kimberlite, which cools rapidly by heat exchange with the atmosphere, remains mostly unaltered preserving its initial permeability, and this then acts as a water pathway into the pipe.

In order to understand the competing influences of both the heat of the reaction and the cooling due to water influx, we performed a numerical experiment for purely conductive cooling of the pipe with no water influx and no serpentinisation. The simulation shows that for conductive cooling, the temperature drops below *T*_*α*_ after 1,260 years, which is actually faster than for the case of water influx. This apparent contradiction is explained by the release of the exothermic heat of reaction of serpentinisation that leads to slower cooling of the kimberlite and a prolonged interval of serpentinisation. We will discuss the relative importance of different processes on the energy budget later.

**Fig. 5:**
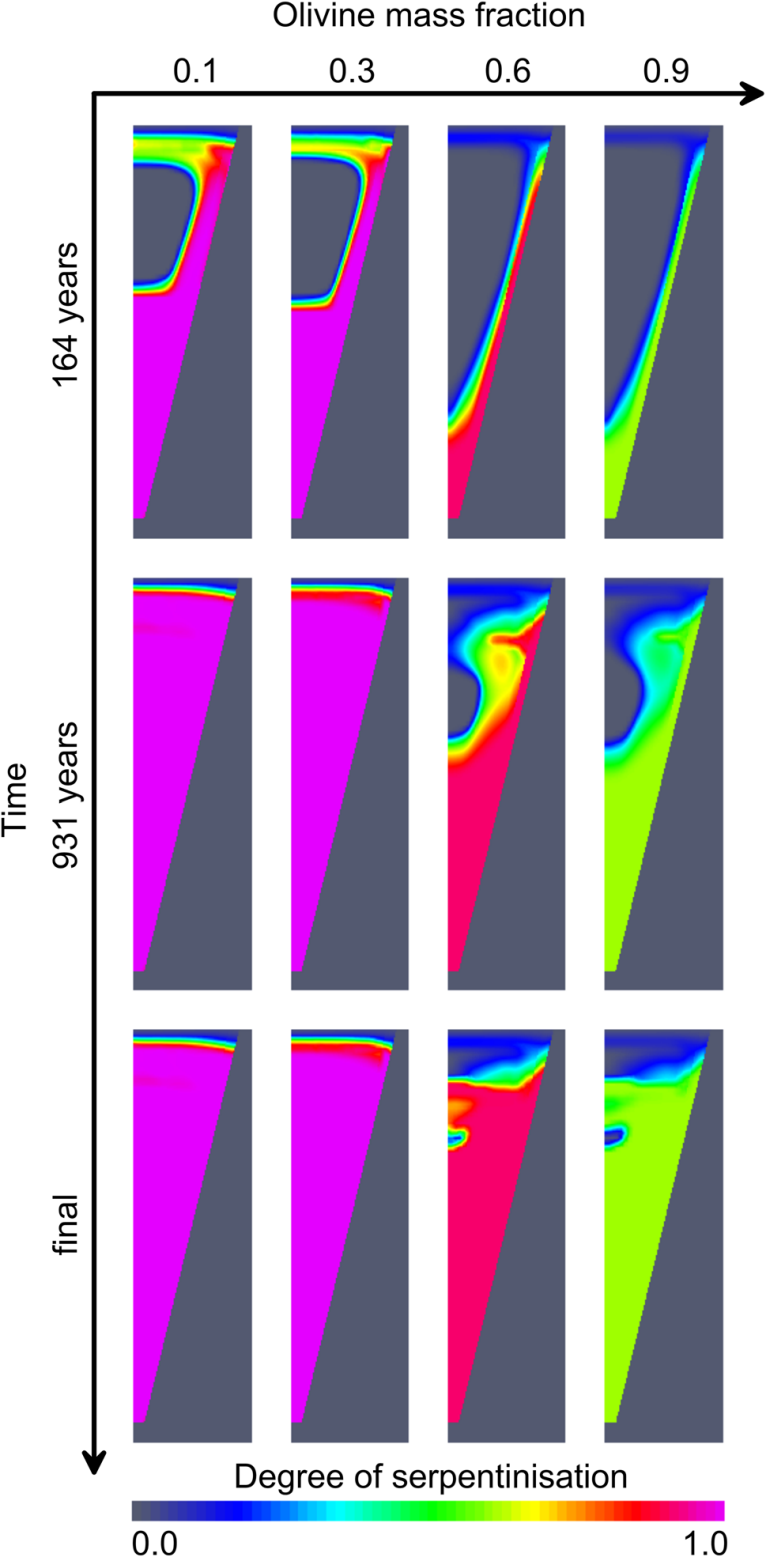
Time snapshots of the degree of serpentinisation for different proportion of olivine in the matrix of the kimberlite. *y* is the proportion of olivine present (*y* = 0—no olivine and no serpentinisation; *y* = 1—100 % olivine). The *red colour* represents the position of the water front. Rock permeability is *K*_*B*_ = *K*_*C*_ = 10^−15^ m^2^. Pipe permeability is *K*_*A*_ = 3 × 10^−14^ m^2^. Serpentinisation stops after 383, 492, 1,862, and 1,971 years for *y* = 0.1, 0.3, 0.6, and 0.9, respectively

Figure 5 illustrates the development of serpentine with time for different initial olivine mass fractions in the kimberlite *y*. There is a drastic difference between the cases of low *y* = 0.1, 0.3 and high *y* = 0.6, 0.9 olivine content. In the first case, the serpentinisation of the olivine leads to relatively small reduction of the porosity and, thus, permeability. In the case of high olivine content, a low permeability layer is formed on the border between the kimberlite and the host rocks dramatically reducing water flux into the pipe. The pipe cools down mostly by conduction. Zones of fresh olivine remain in the body of the pipe. In the case of *y* = 0.9, only approximately 60 % of the initial olivine can be serpentinised because the reaction stops when the porosity of the system becomes negligibly small ending water flux into the deposits. Serpentinisation stops after 383, 492, 1,862, and 1,971 years for *y* = 0.1, 0.3, 0.6, and 0.9, respectively.

**Fig. 6:**
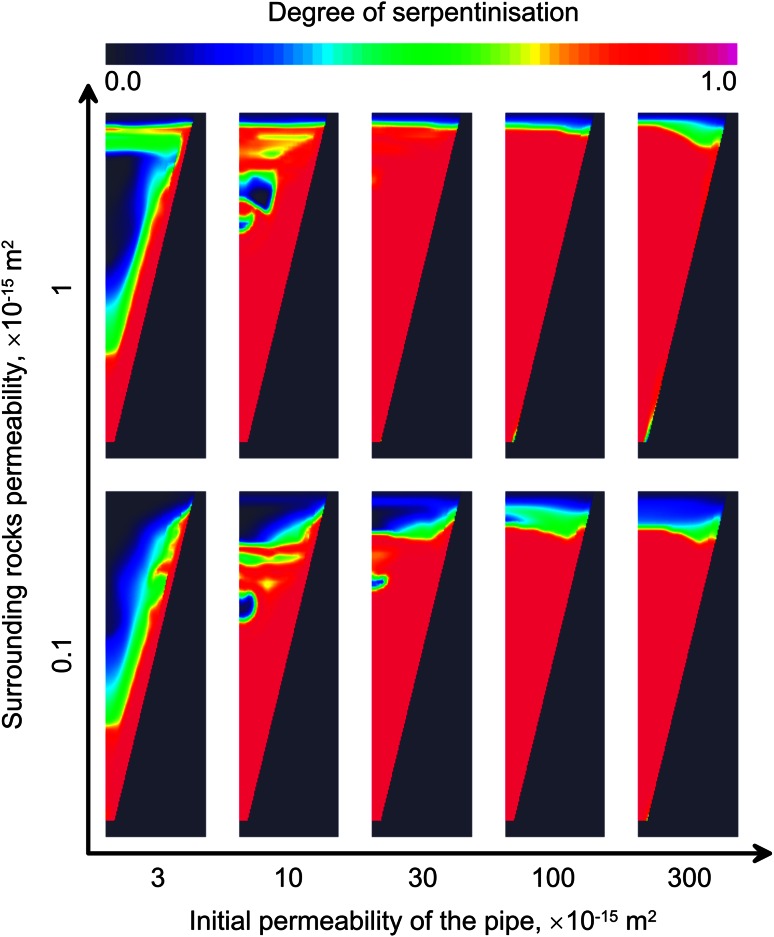
Distribution of the serpentinite in the kimberlite after reaction stops at the temperature of 130 °C. On the *horizontal* axis, the permeability of the host rock increases to the *right*. Complete serpentinization of the kimberlitic body is possible when both kimberlite and host rock are highly permeable

Figure 6 shows distribution of the serpentine in the kimberlite after reaction stops. Insets from the left to the right are ordered by increasing permeability of the kimberlite (*K*_*A*_). Two values of permeability of the host rocks (*K*_*B*_ = *K*_*C*_) are investigated. On all insets, the final stage of the cooling is shown when the temperature of the whole pipe becomes below the *T*_*α*_. It takes a longer time for low permeability kimberlite to reach this final stage (see Table 2 for details). Where kimberlite permeability is low, the water cannot penetrate deep inside the kimberlite and conductive cooling occurs without serpentinisation leaving the central part of the pipe with fresh olivine. With increasing permeability of the kimberlite, convective cooling now influences heat release, more water penetrates into the pipe, leading to larger volumes of serpenitinised kimberlite, and cooling times are shorter. Complex structures can develop due to the competition between cooling and serpentinisation that leads to significant permeability reduction and release of latent heat of the reaction. Zones of incomplete serpentinisation are developed. These results are not very sensitive to the permeability of the host rock. Even at very high pipe permeability (*K*_*A*_), release of the heat due to serpentinisation significantly increases the time until serpentinisation ceases.

**Table 2:** Duration of serpentinization of the kimberlitic pipe after emplacement (years)

Host rock permeability, *K*_*B*_ and *K*_*C*_ (×10^−15^ m^2^)	Kimberlite permeability *K*_*A*_ (×10^−15^ m^2^)				
	3	10	30	100	300
0.1	1,590	2,030	1,860	1,420	880
1	1,530	2,140	1,590	1,150	600

**Fig. 7:**
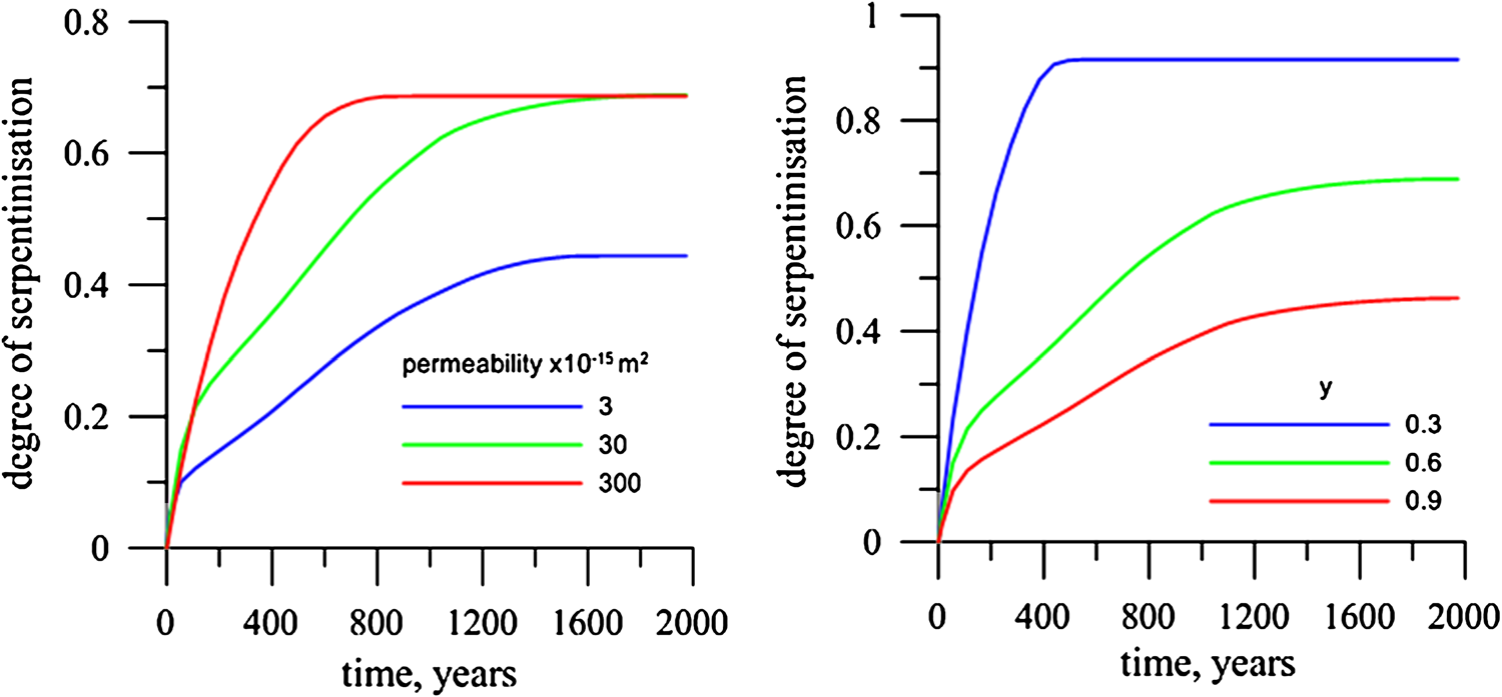
Time evolution of the degree of serpentinization in the pipe for different permeability (**a**) and olivine content (**b**) of the kimberlite. Rock permeability (*K*_*B*_ = *K*_*C*_) is 10^−16^ m^2^

Figure 7 shows the time of serpentinisation cessation for different kimberlite permeability and different initial olivine mass content *y*. In the case of low kimberlite permeability, *K*_*A*_ = 3 × 10^−15^ m^2^, water cannot penetrate deeply in the pipe, and after ~1,400 years, serpentinisation stops leaving nearly 60 % of the olivine fresh. For higher values of *K*_*A*_, only the top part of the body remains unaltered. The time of cessation decreases with increasing kimberlite permeability. For low initial olivine content (*y* = 0.3), nearly all olivine in the pipe becomes serpentinised after a short period of time. For *y* = 0.9, only 30–40 vol% of the olivine is serpentinised at the time of serpentinisation cessation.

**Fig. 8:**
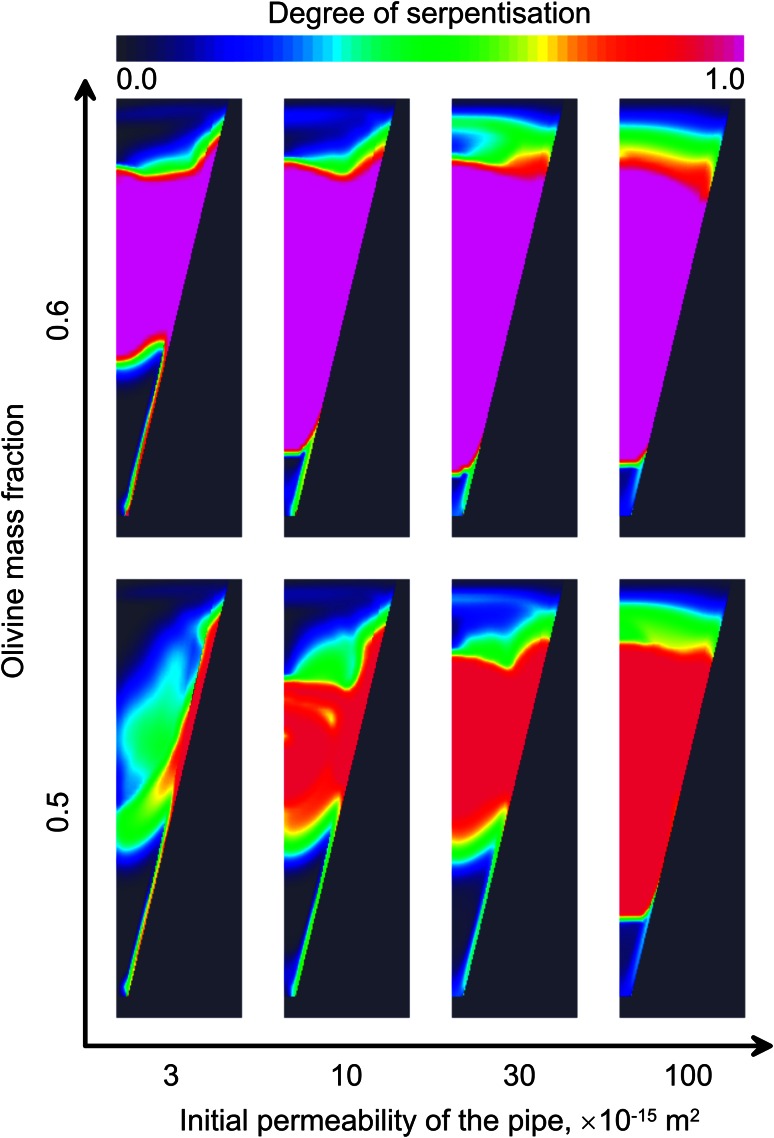
Distribution of the serpentinite in the kimberlite for different initial mass fraction *y* of the olivine in unaltered porous rock and different permeabilities of the kimberlite (*K*_*A*_)

In the case of two-layered host rocks (Fig. 8), the pattern becomes more complicated. Here, we assume that region *C* is impermeable (*K*_*C*_ = 0 m^2^), and the permeability in region *B* is fixed at *K*_*B*_ = 10^−16^ m^2^. The amount of olivine in the kimberlitic matrix, *y*, varies from 0.5 to 0.6, and the permeability *K*_*A*_ changes by two orders of magnitude. For the olivine mass fraction *y* = 0.6, complete serpentinisation is possible in the upper part of the kimberlite, but the accompanying permeability reduction is large so that deeper parts of the pipe cool to temperatures below *T*_*a*_ (i.e. too low for serpentisation) before water is able to reach them. In this case, the upper layer of the kimberlite becomes completely serpentinised except the uppermost part that cools quickly by heat exchange with atmosphere. The water also propagates downward leading to partial serpentinisation of the kimberlite below the boundary between regions *B* and *C*. Significant reduction of permeability in the upper part of the kimberlite prevents water from penetrating downwards quickly enough to cause serpentinisation prior to cooling below *T*_*a*_. Even in the case of extremely large kimberlite bodies, permeability at the base of the pipe remains unaltered.

## Discussion

The numerical simulations demonstrate that ground water flows easily and rapidly into a pipe infilled with hot volcaniclastic deposits. External fluids are drawn in, are heated, and ascend upwards in the centre of the pipe. The models abstract the alteration process by considering serpentisation of olivine as the dominant reaction. The reactions lead to a decrease in porosity and permeability. Because the serpentinisation reaction is typically fast, in comparison with the cooling time scale of the pipe infill, there is a sharp reaction front, dividing into regions with olivine and regions with abundant serpentine at any one time (Fig. 4).

### Cooling time scales and water–rock ratios

Cooling time scales of centuries to a few millennia are calculated for the olivine fraction *y* = 0.6 (Table 2). Serpentisation reduces porosity and permeability leading to suppression of fluid flow. Thus, the flow regime is not like a typical hydrothermal system, where large amounts of fluid circulate freely and greatly reduce cooling times compared to pure conduction. Using our model with constant porosity and no hydration reactions, the pipe cools rapidly (~200 years to reach *T*_*α*_) through conductive and convective cooling. With the hydration reactions, however, cooling time increases by a factor of 10 (see Fig. 5 caption for details) and can exceed the cooling time by pure conduction (1,310 years) due to the counteracting effect of heat generated by the reaction.

We have calculated the total amount of water drawn into and through the pipe. For the parameters in our representative case (Fig. 4), we integrated the flux of water over the side surface of the pipe from time zero to final cooling time to *T*_*a*_. The total mass of water then was divided by the total mass of rock in the pipe at *t* = 0 (later the total mass of the rock changes because olivine gains some water during serpentinisation). The water-to-rock mass ratio is 0.098, and water/olivine ratio is 0.164. Since 73 % of this water is consumed in the serpentisation reaction, there are rather modest amounts of water which flow completely though the system. In contrast, very high water rock ratios are characteristic of major geothermal convective systems that are driven by magmatic heat from large magma reservoirs.

### Geological controls

Kimberlite geology reflects complex multiphase eruptions and depositional events. A wide variety of lithofacies have been recognised, including massive and layered volcaniclastic deposits with diverse genesis and characteristics (Scott Smith 2008; Sparks 2013). Our models show that porosity, permeability, temperature, and olivine content are the main controls on serpentisation. Our results indicate that olivine content, porosity, and permeability of the kimberlite are, in general, more important than the hydraulic properties of the host rock to the extent of serpentinisation, due to the large volume increase of the products of the reaction. Temperature governs the kinetics of the reaction, and our model is specifically for kimberlite deposits emplaced at sufficiently high temperatures for fast reactions. The preservation of fresh olivine in some Fort à la Corne volcaniclastic kimberlites (e.g. Pittari et al. 2008; Scott Smith 2008) likely reflects either their low-temperature emplacement (e.g. <100 °C) during phreatomagmatic eruptions and reworking in marine environments or local lack of water access to parts of the volcaniclastic kimberlites.

Common types of pyroclastic deposits identified in kimberlites include pyroclastic flows, surges, and fallout, as well as massive deposits attributed to gas fluidisation. Breccias, lapilli tuffs, and tuffs indicate a wide range of possible porosities and permeabilities within the pipes, noting that young (non-kimberlitic) pyroclastic deposits typically have porosities in the range 0.2–0.5. Fine-grained, poorly sorted deposits are expected to have lower porosity and permeability, while coarser-grained well-sorted deposits have higher porosity and permeability. In addition, deposits will have different emplacement temperatures influenced by eruption style and abundance of included cold country rock lithic fragments. Facies architecture and the contacts between facies can also influence the pathways of migrating fluids and will therefore affect the progression of serpentinisation throughout the pipe. Even small-scale features such as bedding may influence the fluid pathways and cause local fluctuations in alteration intensity. The models suggest that pervasive serpentinisation is predicted over a wide range of these key parameters. These predictions are, therefore, consistent with observations that many kimberlites are strongly altered volcaniclastic deposits that had high initial porosities.

**Fig. 9:**
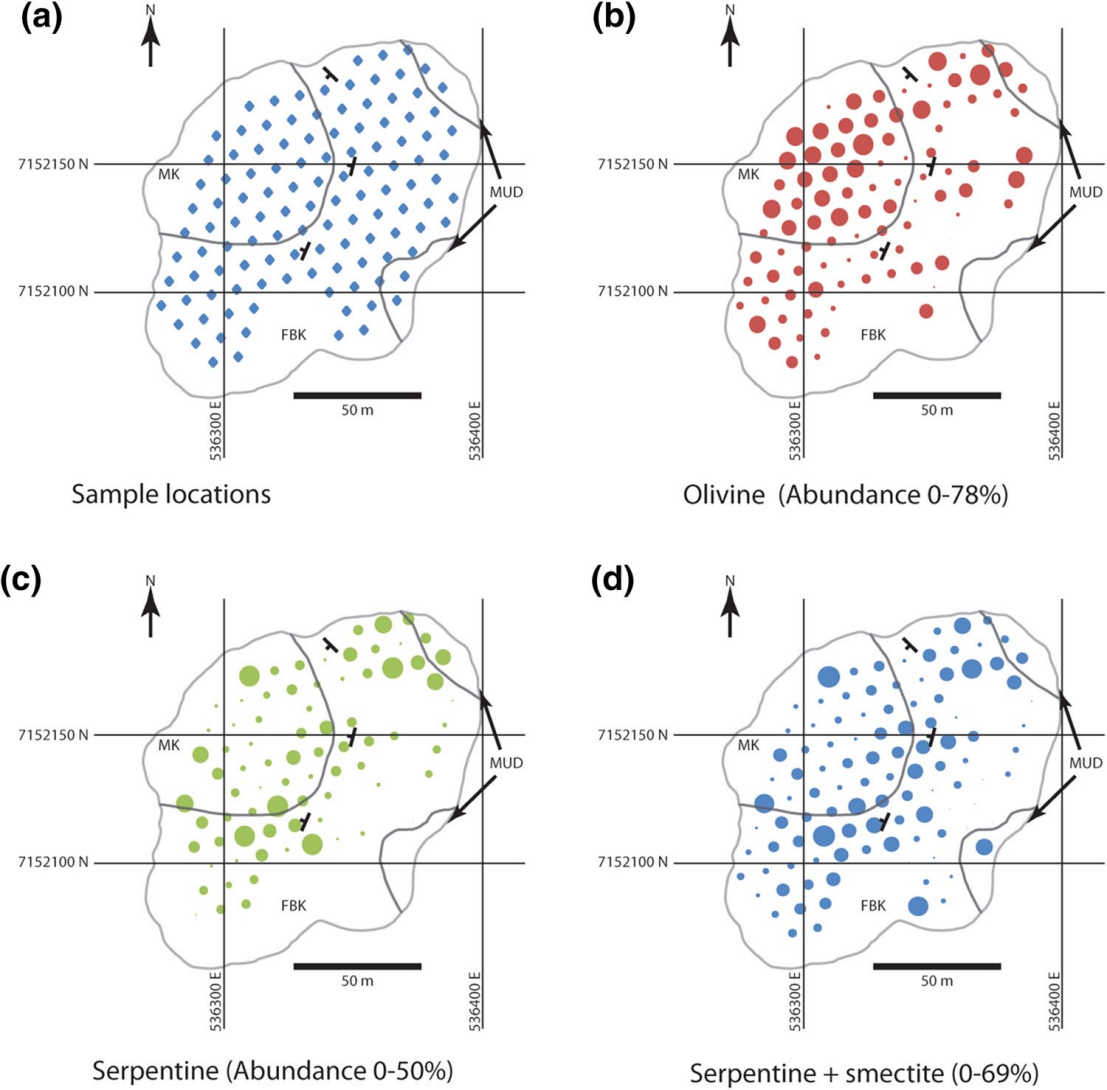
Geological maps of the A418 kimberlite pipe, Diavik Diamond Mine, NWT Canada, showing the geology exposed on the 290 Level bench and the distribution of key minerals. *MK* massive kimberlite, *MUD* mud-rich, olivine poor kimberlite, *FBK* finely bedded kimberlite (see text for further details. **a** Location of the sampling grid for XRD analysis, **b** distribution of fresh olivine ranging from 0 to 78 % based on XRD analysis, **c** distribution of serpentine ranging from 0 to 50 % based on XRD analysis, **d** distribution of serpentine + smectite ranging from 0 to 69 % based on XRD analysis. Note the higher abundance of fresh olivine in the MK, and the enhanced alteration developed in proximity to internal geological contacts as shown by serpentine and smectite abundances

**Fig. 10:**
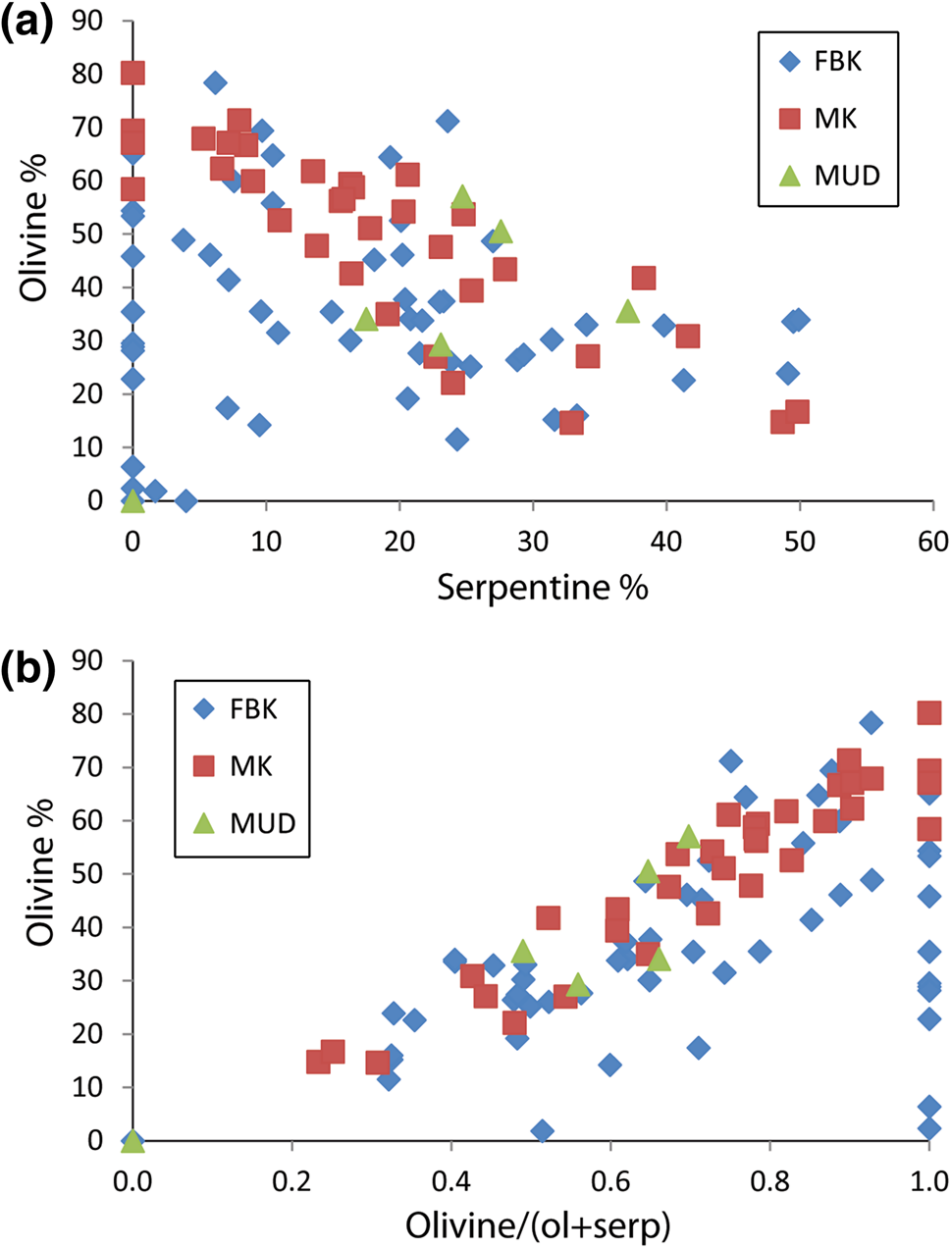
Graphs showing the results of XRD analysis of samples from the three lithologies at the A418 pipe. **a** Plot of olivine abundance against serpentine abundance, **b** plot of total abundance of fresh olivine against the fraction of fresh olivine in the olivine + serpentine component of the rock (a measure of how altered the rock is 1 being fresh and 0 being totally altered), and based on our modelling results, we can translate degree of alteration into a measure of original porosity (*φ*) with complete alteration occurring when the original porosity exceeded 40 %

Here, we give an example of serpentisation observed at the Diavik A418 pipe, NWT, Canada, where deposit-scale variations in the abundance of serpentine and olivine were examined in detail. Over a hundred samples were taken from the 290 m bench in the open pit and analysed for mineral abundance using quantitative X-Ray Diffraction (XRD) by the Saskatchewan Research Council in Saskatoon. Sampling was carried out by Diavik geologists on a grid (Fig. 9a). The three main lithologies within the pipe were included in the sampling as follows: (1) MUD, a massive kimberlitic silt to fine sand with low abundances of olivine macrocrysts (>1 mm, <10 %); (2) MK, a massive, poorly sorted, mudstone clast- and olivine-rich volcaniclastic kimberlite with 20–50 % olivine macrocrysts (which includes altered and unaltered olivine); (3) FBK, a diffusely to well-bedded (mm-to-cm scale bedding) ash-aggregate, and olivine-rich volcaniclastic kimberlite with highly variable olivine macrocryst abundance (20–90 % in individual beds) (Porritt et al. 2012). The abundance of olivine and serpentine measured by XRD in the samples is shown in Fig. 9b, c, and the abundance of serpentine and smectite is seen in Fig. 9d. Smectite is a product of advanced alteration of other components of the kimberlite. The same data are plotted on diagrams of olivine versus serpentine content and olivine versus the ratio of olivine to serpentine plus olivine (Fig. 10)

Although there is considerable overlap, MK has the highest modal abundance of olivine macrocrysts of the three lithofacies and has a higher average olivine/olivine + serpentine ratio than the other deposit types (Fig. 10; 0.71 MK; 0.54 FBK; 0.25 MUD). While poor sorting of the deposit and high original olivine content results in slow water infiltration, complete infilling of the pores with serpentine arrests the serpentinisation process and leads to preservation of fresh olivine. The most serpentinised portions of the MK occur near the pipe walls and in proximity to the contact with the FBK, which are the areas of likely fluid influx into the deposit. The FBK deposits show increased abundance of serpentine in the proximity of the pipe wall and MUD contact in the north of the pipe, and along the contact with the MK in the centre of the pipe, which is consistent with fluid migration along internal boundaries as well as external contacts. These observations are consistent with our expectations that higher porosity and permeability rocks are more susceptible to serpentinisation, and that serpentinisation can localise at the pipe (or unit) margins. Olivine-rich rocks are more strongly serpentinised, provided there is enough porosity for the excess volume of solid products to precipitate. Each of the lithofacies shows a wide range of olivine and serpentine (Fig. 10) with a broad anti-correlation. The data are consistent with initial porosities varying between 40 % and near zero.

A decrease in serpentinisation at depth has been noted in several pipes, for example at the Fox pipe in the NWT, Canada (Porritt and Cas 2009), and Gahcho Kué pipe (Hetman et al. 2004). At Fox pipe, the F1, a massive, poorly sorted kimberlite deposit, pseudomorphed olivine grains occur with fresh glassy cores, whereas at higher levels, more intense alteration has completely pseudomorphed the olivine grains. This trend is consistent with less infiltration of water at depth where country rocks are less permeable and cooling is faster due to the pipe being narrower, as predicted in the models (Fig. 7).

The alteration model results provide insights into two related issues concerning the origin of coherent kimberlitic rocks and transitional rocks between volcaniclastic and these coherent rock types. The latter have widely been interpreted as hypabyssal in origin, but are now increasingly interpreted as densely welded, high-temperature volcaniclastic rocks (Brown et al. 2008a). Coherent kimberlite is particularly common in the root zones of kimberlites, although they can occur at any level. In many pipes, such as the Gahcho Kué pipe, NWT Canada (Hetman et al. 2004), textural variations between volcaniclastic rocks and coherent kimberlite are described and termed transitional. One of the features of the transitional volcaniclastic kimberlite is partially serpentinised olivines, while tuffisitic kimberlite contains completely pseudomorphed olivine and coherent kimberlite commonly contains abundant unaltered olivine (Hetman et al. 2004; Brown et al. 2008a; van Straaten et al. 2011). The lack of serpentisation at depth is readily interpreted as the consequence of rapid densification of high-temperature volcaniclastic kimberlite that reduces porosity and permeability preventing infiltration of water. The common occurrence of coherent or coherent-like kimberlite in root zones is attributed to factors such as late-stage influx of high-temperature kimberlite magma at the base of the pipe (Gernon et al. 2012) or welding of pyroclastic kimberlite (Brown et al. 2008a), both would have low porosities and permeabilities and, hence, result in the sluggish infiltration of external water.

### Source of fluids

Another controversy is the origin of the water involved in serpentisation. The opposing viewpoints of a magmatic fluid and external (ground water sources) are discussed by Mitchell (2013), Sparks (2013), and Giuliani et al. (2014) and are not repeated here. Mitchell (2013) presents ion probe data for δ^18^O in serpentines which have typically positive values in the range >0 to +6.3 with most values exceeding +3. He infers that these data preclude involvement of meteoric water with typically negative δ^18^O. Part of Mitchell’s argument is that water/serpentine ratios cannot have exceeded 0.8 and “that there was not a significant volume of low temperature water as a cause of serpentisation”. We agree with his conclusions about low water–rock ratios, which are predicted by our model (≪0.8). However, water influx slows and then ceases during serpentinisation due to the reduction in porosity and permeability. Just enough water is used up to convert the olivine to serpentine. Water/serpentine ratios are consequently low and result in small changes of δ^18^O.

Depending on the reaction, the change is between 10 and 27 % from the initial olivine value towards the water value and depends on whether oxygen in the original olivine is immobile or there is exchange between the reaction products. For example, in reaction 1, using δ^18^O = −6 for meteoric water and δ^18^O = +6 for olivine, the serpentine will have δ^18^O = +4.8 in the immobile case and δ^18^O = +2.8 with exchange. Furthermore, an initially light δ^18^O fluid that exchanges with the kimberlite matrix will have its own oxygen isotope composition modified, to become increasingly positive, due to the high proportion of matrix relative to circulating fluid. Accordingly, the shift from mantle δ^18^O may be even smaller or even negligible depending on the evolution of the fluid. This is consistent with the observations, as the majority of the oxygen in the serpentine originates from the olivine.

There is evidence for external fluids infiltrating into kimberlites in the Yakutian kimberlite province, Russia, where the kimberlites were emplaced into a thick sequence of sedimentary rocks, including carbonates and evaporites, and are associated with brines (Kopylova et al. 2013). The Mir kimberlite is largely serpentinised, but contains groundmass halite and shortite, which may reflect the salty character of the ground waters in the host rock limestones, marls, and dolomites. In the case of the International’naya kimberlite, the kimberlite is elevated in Na and S where there are halite and anhydrite and gypsum beds, respectively, adjacent to the kimberlite. In these regions, the olivine is fresh. The Yakutian kimberlite province includes the unusual Udachnaya kimberlite, where fresh olivines are preserved in volcaniclastic kimberlite with a groundmass containing abundant halides and alkali carbonates (Kamenetsky et al. 2004). This kimberlite has avoided serpentinisation. Kopylova et al. (2013) observed that: “The localization of the highest abundances of Na–K–Cl–S-bearing minerals in the Udachnaya East kimberlite at a depth interval that correlates across three magmatic phases of kimberlites and coincides with the roof of the halite-bearing country rock and an aquifer carrying anomalously Na-rich brines”. Kopylova et al. (2013) also present stable isotope data consistent with interactions between kimberlite and host rocks and brines. The striking feature of these three cases is that the mineralogical characteristics of the matrix of the kimberlites vary in broadly horizontal zones that coincide with wall rocks and different composition brines. In the case of Udachanaya pipe, the matrix mineral assemblages span three geological units emplaced at different times. The localisation of abundant alkali carbonate and halide minerals within the kimberlite matrices adjacent to brine aquifers and carbonate host rocks can be explained by groundwater infiltrating into the kimberlite.

Brine alteration might be caused by post-emplacement low-temperature infiltration of regional brines, but this hypothesis is not supported by observations of high-temperature olivine inclusions. Kamenetsky et al. (2004) documented the presence of sodalite and inclusions in olivine containing halide-carbonate mineral assemblages, which equilibrated at temperatures of well above 400 °C. They attribute the halides and alkali carbonates to residual melts and magmatic fluids to the kimberlite magma. However, the model of infiltration of brine into hot kimberlitic volcaniclastic deposits can also explain these observations and is analogous with our model of water infiltration discussed above. Kopylova et al. (2013) thought that these temperatures were too high to support alteration. However, brines can infiltrate and react with kimberlite at any temperature at or below the emplacement temperature of the kimberlite. Indeed, at temperatures of 600 °C, it is possible to envisage heated infiltrating brines reacting with kimberlite, the formation of high-temperature melts of natrocarbonatite, and brine forming in pore spaces and then crystallizing out assemblages with igneous textures. Brines saturated in CO_2_ become acidic and are strongly reactive with calcite (Lamy-Chappuis et al. 2013), and dissolution of original igneous calcite in the kimberlite might be an additional factor with creation of secondary porosity. If these processes occur above 400 °C and infill pore space, then water is prevented from accessing the kimberlite after the temperature declines and, thus, explains preservation of fresh olivine. Although physical incorporation and perhaps assimilation of carbonates and evaporites into kimberlite melts prior to emplacement likely played a role in the characteristics of the Yukatian kimberlites (Kopylova et al. 2013), these processes are not mutually exclusive. High-temperature late fluids exsolved from a magma contaminated with evaporates and carbonates may be difficult to distinguish from fluids generated by infiltration and heating of brines to high temperatures.

### Model assumptions and caveats

The model is necessarily a simplification of what might happen in natural systems, so we briefly consider relaxing some of the assumptions. Any model of a geological process has to make simplifying assumptions to be tractable, and simplified models have the benefit of being easier to interpret than more complex models, which attempt to include many possible effects. We briefly comment qualitatively on relaxing some of the assumptions in the model.

Reactions among hydrothermal fluids and kimberlite constituents involve more than olivine and serpentine. Although some reactions, such as the formation of magnetite from the Fe component of olivine, may reduce the solid volume, the majority of products are hydrated minerals, such as talc, chlorite, and smectite. The products of more complex reactions will not change the model results. Multiple generations of different serpentine polymorphs are also a consequence of changing temperature and conditions in the circulation of fluids as the pipe fill cools. Mitchell (2013) draws attention to the sequence of serpentine formation with decreasing temperature in serpentine veins in oceanic peridotites (Andreani et al. 2007) and remarks that this is the same sequence as observed in kimberlites. We concur, but draw a different inference that this similarity is consistent with the same process in both cases, namely progressive alteration from external water. If swelling clays form at low temperature, the kimberlite can become a self-sealing system resistant to further infiltration.

There will be porosity variations within the pipe reflecting geological complexities. Lower porosity, finer-grained volcanic, and sedimentary deposits resides in the uppermost crater zones and forms a low permeability cap. In this case, the internal pressure in the pipe might be expected to become high and so ground water flux rates into the pipe would be slower. However, this should make a major difference. The model predicts that the process is largely the pipe absorbing water from its surrounding and the flow through is modest. In the case where water flows in but cannot flow out, the system will move to a balance of the hydrostatic pressure inside and outside of the pipe. However, this balance can only be achieved for water when the temperatures inside and outside of the pipe are the same. Thus, we anticipate an early stage dominated by inward flow of water and a later convective stage when the main driving force comes from the difference in temperature between water inside and outside of the pipe. This later stage should involve outward flows of hot fluid as well as inward flows of cold water. Country rocks might be altered in the outflow areas.

We have assumed a closed system with only local mobility of Si and Mg to facilitate the serpentinisation reaction. However, high pH fluids emerging from ocean flow hot springs associated with serpentisation (e.g. Kelley et al. 2007) can carry high concentrations of major elements such as Ca^2+^ and Mg^2+^. Solute transport will lead to complexities, including re-distribution and quantitative removal of major components. Models that include such complexities are avenues of research, but they should not introduce any fundamental new aspect of the process. Likewise, geological complexities, such as local aquifers and faults, could be included in site-specific modelling studies.

We have only considered conversion of olivine to serpentine. An unmodelled effect, however, is dissolution of minerals. Primary igneous carbonate (typically calcite) has been found in many hypabyssal kimberlites, and models of kimberlite magmas suggest carbonate-rich residual melts (Russell et al. 2012). Textural evidence (Sparks et al. 2009) indicates that calcite and other Ca-rich phases such as apatite, in hypabyssal kimberlite, are dissolved in the presence of serpentinising fluid, an observation consistent with the high Ca content of fluids generated during ocean ridge serpentisation (Kelley et al. 2007). However, primary carbonate is typically absent in pipe-filling volcaniclastic kimberlites, which may be explained by dissolution during serpentisation. Dissolution of calcite and other Ca-rich minerals creates secondary porosity and enhances the prospects for incoming fluids to serpentinise olivine.

Our model considers only high-temperature kimberlite deposits. However, some volcaniclastic kimberlites were likely emplaced cold resulting in a much reduced rate and degree of serpentinisation. Involvement of water in the eruption, either from surface water or ground water from high permeability country rocks, or deposition of the volcaniclastic kimberlite onto the surface outside of the pipe (and subsequent cold resedimentation into the pipe) both result in low-temperature deposits within the pipes. Low temperature may be the reason that olivine is typically well preserved in many of the Fort a la Corne kimberlites, which are attributed to phreatomagmatic eruptions in a marine environment (Pittari et al. 2008).

## Conclusions

Our model shows that the timescales of cooling of a kimberlite pipe, from emplacement temperatures through the serpentinisation temperature window to *T*_*e*_, and the timescale of infiltration of external water, which drives the serpentinisation reaction, are comparable. The rate of infiltration of water into the pipe, and consequently the ability of water to react with the kimberlite before it cools below the serpentinisation window, is greatly influenced by the deposit porosity, permeability, and olivine content. Deposits with high initial porosity, permeability, and olivine content are more susceptible to serpentinisation, with olivines being pseudomorphed and the excess volume of serpentine formed during the reaction infilling the pore space. High degrees of serpentinisation along initial fluid pathways, such as pipe margins or internal facies contacts, may occlude porosity preventing further ingress of water, thus preserving fresh olivine in the pipe interiors. Post-emplacement serpentinisation of volcanic deposits within a kimberlite pipe therefore can result from the rapid infiltration of external water and reaction with olivine and other kimberlite components.
